# First Insight Into Multiple Paternity in the Potato Cyst Nematode *Globodera pallida* Facing Masculinizing Plant Resistance

**DOI:** 10.1002/ece3.73885

**Published:** 2026-06-29

**Authors:** Magali Esquibet, Océane Lechevalier, Nathan Garcia, Sylvain Fournet, Eric Grenier, Josselin Montarry

**Affiliations:** ^1^ IGEPP, INRAE, Institut Agro Univ Rennes Le Rheu France; ^2^ ANSES Nematology Unit, Plant Health Laboratory Le Rheu France

**Keywords:** experimental evolution, *Globodera pallida*, multiple paternity, plant resistance, polyandry

## Abstract

Polyandry is a common reproductive behaviour in nature, and it was observed in several plant parasitic cyst nematodes. For the potato cyst nematode *Globodera pallida*, polyandry is assumed but has never been demonstrated and quantified. Since the potato resistance used to control this nematode, i.e., the resistance conferred by GpaV from *Solanum vernei,* acts by masculinizing populations, and because polyandrous mating is more frequent in male‐skewed populations, the level of polyandry can be expected to decrease during the nematode adaptation process to potato resistance. Aims of this study were thus to determine whether polyandry occurs in *G. pallida* and to explore the polyandry evolution during the adaptation process to the potato resistance. Using *G. pallida* lineages obtained from experimental evolution on susceptible and resistant potato cultivars, we explored and quantified the genetic evidences of multiple paternity within cysts by genotyping juveniles using microsatellite loci. Results clearly highlighted multiple paternity in *G. pallida* and showed that 100% of females were polyandrous, with an average of seven fathering males. Contrary to our expectations, the frequency of polyandrous females and the female mating rate with different males, estimated from the minimum number of fathers, appeared to remain stable throughout the adaptation process to a masculinizing resistance. The level of polyandry highlighted here may represent an important parameter to consider in demo‐genetic models designed to compare nematode population control strategies.

## Graphical abstract

This study demonstrated multiple paternity in the potato cyst nematode *Globodera pallida*: all females were polyandrous. Moreover, the female mating rate with different males appeared to remain stable, i.e., an average of seven fathering males, throughout the adaptation process to a masculinizing potato resistance.

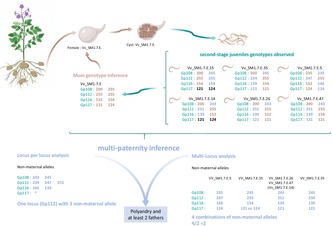

## 1 Introduction

Potato cyst nematodes (PCN) comprise two species, *Globodera pallida* (Stone) and *G. rostochiensis* (Woll.), both of which have significant global economic impacts representing a threat to potato crops (Jones et al. 2013). Difficult to manage, PCN are classified as quarantine pests in many countries, which places severe constraints on potato production. Strict sanitation measures are essential to prevent the spread of PCN and protect pest‐free potato fields from an introduction of these nematodes. In the event of a potato cyst nematode infestation, the use of control methods is mandatory and mostly includes the use of plant host resistance and/or a combination of other strategies. Therefore, resistant potato cultivars, which do not allow cyst nematode reproduction, have become a key environmentally friendly and effective component of integrated management programmes.

PCNs are diploid organisms with obligate sexual reproduction. The cyst is the survival stage of the nematode and serves as an effective means of passive dispersal (Alenda et al. 2014; Esquibet et al. 2024). Protected within the cyst, PCN eggs can survive in the soil for up to decades (Turner 1996). When the presence of roots of a nearby potato triggers their hatching, the infective juveniles (J2) emerge from the cyst and move actively through the soil in search of a suitable root host. Once inside the root, juveniles release numerous secretions that manipulate the plant's defences and induce the formation of a specialised feeding site, the syncytium. Subsequently, juveniles differentiate into either males or females. Adult males leave the root to seek females for mating, so they no longer feed. In contrast, females remain sedentary and require a substantial nutrient supply to support reproduction. When the PCN females die, after completing oviposition, their bodies wall cutinised to form cysts that protect the newly produced eggs.

Polyandry (i.e., the fact that females mate with multiple males) is a very common reproductive behaviour in nature; it was observed in the 14 major taxonomic groups of animals investigated by Taylor et al. (2014), from sea spiders to mammals. In plant‐parasitic cyst nematodes, it is generally accepted that females are polyandrous and males polygynous. The use of biochemical markers to determine the parentage of progeny has directly demonstrated that multiple mating, resulting in multiple paternity, occurs in the cyst nematode *Heterodera glycines* (Triantaphyllou and Esbenshade 1990). Conversely, there is no direct evidence that multiple mating occurs in *G. rostochiensis* and *H. schachtii* . For these two cyst nematodes, polyandry has been explored by indirect observations (Jones 1967; Green et al. 1970). Males of *G. rostochiensis* and *H. schachtii* appear to be able to inseminate several females (Green et al. 1970), and females may be inseminated by up to six or seven males (Jones 1967). In *G. pallida* , the sister species of *G. rostochiensis*, polyandry has been assumed to occur, but has *never* been proven or quantified.

In Europe, the resistant QTL *GpaV*_*vrn*_, derived from the wild species *Solanum vernei* , has been the most frequently used QTL by potato breeders. Widely used in resistant European potato cultivars, *GpaV*_*vrn*_ confers partial resistance to *G. pallida* (Schaveling, te Molder, et al. 2026) and acts by masculinizing nematode populations (Fournet et al. 2013). The mechanism of sex determination of PCN (Trudgill 1967; Mugniéry and Fayet 1981; Mugniéry 1982) is clearly driven by the quality of the syncytium and the availability of nutrients. Juveniles moult into either adult female when nutrient levels are high or adult male when nutrient levels are low (Schaveling, van de Ruitenbeek, et al. 2026). *Globodera pallida* juveniles are able to migrate into the roots of resistant cultivars, but they fail to elicit the formation of a functional syncytium. As a result of insufficient nutrition, *G. pallida* juveniles are unable to develop into females within the roots of resistant cultivars, leading to the production of a high proportion of males (Jones and Parrott 1965). Masculinizing resistances, which prevent female development and thus the formation of new cysts containing eggs, represent a promising option to control *G. pallida* populations. However, its intensive deployment has led to the emergence of virulent populations that can overcome the resistance. Indeed, virulent *G. pallida* populations have been reported in several locations in Europe (Niere et al. 2014; Mwangi et al. 2019; Grenier et al. 2020; Schaveling, te Molder, et al. 2026). Virulent nematodes are able to induce the development of nutrient‐rich syncytia allowing the development of both males and females. *Globodera pallida* populations, introduced to Europe from South‐America (Evans et al. 1975; Grenier et al. 2010), were probably composed of a mixture of virulent and avirulent individuals, and the deployment of resistant potato cultivars in Europe has exerted selection pressure on nematode populations, leading to a shift in the proportion of alleles of virulence. Virulent *G. pallida* populations have been similarly obtained in different experimental evolutions performed under controlled laboratory conditions (Fournet et al. 2013; Varypatakis et al. 2020; Lechevalier et al. 2025; Schaveling, te Molder, et al. 2026). On different resistant potato cultivars harbouring the resistant QTL *GpaV*_*vrn*_, avirulent nematode lineages produced a high rate of males, highlighting the efficiency of masculinizing resistance. However, after several generations of selection on those cultivars, virulent evolved lineages produced significantly more females than avirulent populations. The proportion of adult males in the population consequently varies over time during the adaptation process, with a decreasing proportion of males.

Adult sex ratio can play a central role in mating systems with implications for breeding behaviour (Székely et al. 2014). For example, some bird (Grant and Grant 2019; Liker et al. 2014) and reptile species (Pipoly et al. 2023) have been found to be more frequently polyandrous in male‐skewed populations. Assuming each *G. pallida* female has the capability to mate with more than one male, higher frequencies of polyandrous females can be expected in an avirulent population, compared to virulent populations where males are relatively fewer.

Given that *G. pallida* ‐resistant potato cultivars are masculinising and considering the potential effects of male‐skewed populations on polyandry, this study aimed (i) to determine whether polyandry occurs in *G. pallida* , (ii) to estimate the number of fathers contributing to the offspring contained within a cyst, and (iii) to explore the polyandry evolution during the adaptation process to the potato resistance. We hypothesise that the degree of polyandry could decrease over time during the adaptation process. For this purpose, we searched for genetic evidence of multiple paternity within cysts by genotyping juveniles at seven microsatellite loci. The study was performed using cysts from different stages of the experimental evolution conducted by Lechevalier et al. (2025) during 10 successive generations on the resistant cultivar Iledher (virulent lineage) and on the susceptible cultivar Désirée (avirulent lineage).

## 2 Materials and Methods

### 2.1 Globodera Pallida *Lineages*

The virulent and avirulent *G. pallida* lineages used in this study were obtained through an experimental evolution approach performed under greenhouse conditions (Lechevalier et al. 2025). Among the different lineages of Lechevalier et al. (2025), we chose to retain the virulent nematode lineage Vv_SM1 and the avirulent nematode lineage D_SM1. These two lineages were obtained by rearing a natural nematode population collected near St‐Malo in France during 10 successive generations (i.e., 10 years, as *G. pallida* performed one generation per year under European conditions) on either the resistant potato cultivar Iledher (Vv_SM1) or the susceptible cultivar Désirée (D_SM1). By inoculating 10 juveniles in a Petri dishes test, the avirulent lineage (D_SM1), reared on the susceptible cultivar Désirée, produced an average of 67.2% females on this cultivar, but only 1.5% females on the resistant cultivar Iledher. This initially low proportion of females increased over the 10 successive generations on the resistant cultivar Iledher to reach 40% of females at the tenth generation in the virulent lineage (Vv_SM1). Conversely, the number of males decreases during the selection process.

Starting with the fourth generation, we sampled five cysts (named from A to E) from the batch of cysts obtained each year and stored at 4°C. We did not have any cysts available for the first 3 years because the number of females produced during these years was very low, and all the cysts were used for the nematode multiplication. Thirty‐five Vv_SM1 cysts (5 cysts × 7 generations, from the fourth to the tenth generation [sample's names from Vv_SM1.4 to Vv_SM1.10]) and 20 D_SM1 cysts (5 cysts × 4 generations, from the fourth [D_SM1.4], the sixth [D_SM1.6], the eighth [D_SM1.8], and the tenth [D_SM1.10] generation) were collected and used for DNA extraction.

### 2.2 Microsatellite Genotyping

We searched for genetic evidence for multiple paternity within cysts, each cyst containing the offspring of a single female, by genotyping 40–48 s‐stage juveniles (J2) isolated from each cyst. The genotyping protocol was the same as in Montarry et al. (2015, 2019), but we selected only one of the two multiplex panels. This panel is composed of markers, especially Gp108 and Gp116, for which allelic richness (i.e., the mean number of alleles per locus) is high. Total genomic DNA was extracted from single individual juveniles as described by Boucher et al. (2013). All individuals were genotyped at seven microsatellite loci: Gp108, Gp112, Gp116, Gp117, Gp122, Gp126 and Gr67. Genotyping was performed on ABI 3730XL sequencer (Applied Biosystems) at the Gentyane INRAE platform. Allele sizes were identified using the automatic calling and binning procedure of GeneMapper v 5.0 (Thermo Fisher Scientific) and completed by a manual examination of irregular results. An unbiased estimate of gene diversity (*H*_nb_ according to Nei 1978) was computed for each cyst using GENETIX 4.05.2 (Belkhir et al. 2004).

### 2.3 Analysis of Paternity

**FIGURE 1:**
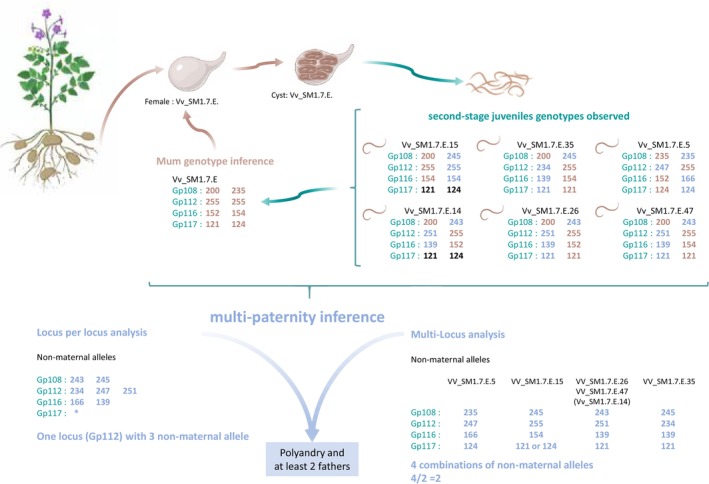
A simplified figure illustrating how a minimum number of fathers can be manually inferred from six juveniles and four microsatellite markers. Maternal alleles were deduced from all the alleles found in the offspring of the cyst VV_SM1.7.E. For each juvenile, the maternal allele and the non‐maternal allele are indicated in brown and blue respectively (in black, alleles with an undetermined origin). Using the ‘locus per locus analysis’, we deduced multiple paternity: More than two non‐maternal alleles (3) occurred in at least one locus (1/4). Using the ‘multi‐locus analysis’, four possible non‐maternal alleles combinations were identified and based on the diploidy, a minimum of two fathers was deduced from this set of five juveniles.

Analysis of paternity was initially checked manually by evaluating the number of alleles per locus (locus per locus analysis) and the number of multi‐locus genotypes (multi‐locus analysis) detected on each cyst (Figure 1). These procedures are based on the assumptions of Mendelian inheritance and independence of the analysed loci. As a first step, we identified the maternal alleles, from all the alleles found in the offspring of a cyst. If a juvenile is homozygous at a given locus, the mother necessarily carries this allele. Thus, in Figure 1, because the juvenile Vv_SM1.7.E.5 is homozygous at the locus Gp117 (124/124), its mother carries the allele 124. The juveniles Vv_SM1.7.E.35, Vv_SM1.7.E.26, and Vv_SM1.7.E.47 are also homozygous at this locus and carry the allele 121. Therefore, the mother of these four juveniles is necessarily heterozygote and possesses both alleles 121 and 124. This example is straightforward. In the absence of a homozygous juvenile, the maternal alleles are more difficult to identify and they can be inferred by deduction through the analysis of all offspring genotypes, since maternal alleles at a given locus are shared by all juveniles. We deduced multiple paternity if more than two non‐maternal alleles occurred in at least one locus among the offspring (locus per locus analysis). Then a minimum number of fathers was inferred from the number of multi‐locus genotypes identified on each cyst (multi‐locus analysis). For each juvenile and at each locus, at least one allele was expected to be identical with one of the two alleles of the mother. This allele was subtracted to deduce the allele of the father (Figure 1). When a heterozygous juvenile had the same genotype as its heterozygous mother, maternal alleles could not be strictly identified and subtracted as illustrated in Figure 1. The inferred non‐maternal alleles of juvenile Vv_SM1.7.E.14 at locus Gp117 is either 121 or 124. We then evaluated the number of possible combinations of paternal alleles. In the example of Vv_SM1.7.E.14 at the Gp117 locus, the combination of non‐maternal alleles in this juvenile is considered equivalent to that of juveniles Vv_SM1.7.E.26 and Vv_SM1.7.E.47, whose non‐maternal allele is 121. However, it could also have been considered equivalent to that of juveniles whose non‐maternal allele is 124. Based on the diploidy, a minimum number of fathers contributing to the offspring contained within a cyst was deduced by dividing the number of paternal multi‐locus genotypes by two and rounding the result down to the nearest whole integer.

The data were also analysed through the software Colony v2.0.7.0 (April 25, 2023) downloadable from the website http://www.zsl.org/science/research‐projects/software/. Colony is a computer programme implementing a likelihood method and two pairwise likelihood methods to assign/infer parentage, sibship and clonemates (duplicates) among individuals using their multi‐locus genotypes. In brief, the method which accepts missing data assumes a sample of descendants and assigns them (clustered) to paternal families and maternal families. The current model accounts for deviations from Hardy–Weinberg equilibrium but inbreeding (due to mating between close relatives or to population sub‐structure) can also be taken into account. Results from Colony mainly include: (i) Full and half sibship assignments among a sample of descendants, (ii) Genotype inference at each locus of each offspring and (iii) Genotype inference at each locus of each parent. Contrary to the previous analysis, Colony has the advantage to also include possible genotyping errors at each locus of each offspring. It refined allele frequency estimates using the inferred relationships, and also refined estimates of genotyping error rates at each locus. We used the default setting and all runs were performed with unknown maternal genotypes. We provided Colony with a set of allele frequencies calculated for both lineages at each generation (i.e., data of the five cysts being pooled) in order to calculate the likelihood of a configuration.

### 2.4 Statistical Analyses

All statistical analyses were performed using the R software version 4.1.2 (R Core Team, 2021).

The comparison of the three methods (locus per locus analysis, manual multi‐locus analysis, Colony multi‐locus analysis) allowed us to determine the minimum number of males which have mate each female (i.e., each cyst). We used a paired *t*‐test to assess whether the results from the manual multi‐locus analysis differed from those obtained using Colony's multi‐locus analysis.

For the results from the Colony analysis, normality and homogeneity of variances were checked using the Shapiro–Wilk and the Leven tests, respectively. For each *G. pallida* lineage (*Vv_SM1 and D_SM1*), the generation effect was tested for the minimum number of males using a one‐way anova. The potato cultivar effect was tested for the fourth, sixth, eighth, and tenth generation using one‐way anova tests. Moreover, Pearson linear correlations were performed for each lineage.

## 3 Results

### 3.1 Genetic Diversity

Among the seven microsatellite loci (Gp108, Gp112, Gp116, Gp117, Gp122, Gp126 and Gr67), six markers were retained; Gr67 was not conserved because it appears fully monomorphic. To minimise allele reading errors, we introduce missing data when the allele sizes were uncertain. Individuals with more than two missing data across the six loci were removed from all analyses. Cysts D_SM1.6.A and Vv_SM1.8.C, which had respectively an unacceptable amount of missing data for the most polymorphic marker Gp108 and across all loci, were also removed from the analyses. The median number of genotyped juveniles per cyst was 39 and the two cysts having the lowest number of genotyped offspring (*n* = 33) came from both the avirulent and the virulent lineages (D_SM1.4.B *and Vv_SM1*.4.C) (Table 1).

**TABLE 1:** Number of genotyped individuals (*N*) and number of individuals without missing data (*n*) per cyst, genetic diversity (*H*_nb_) and minimum number of fathers contributing to the offspring contained within a cyst as estimated by (a) Locus per locus analysis: Initial multi‐paternity inference and (number of loci with three or more non‐maternal alleles), (b) Multi‐locus analysis: Minimum number of fathers based on the number of multi‐locus genotype combinations, (c) Colony: Number of fathers based on the number of multi‐locus genotypes inferred using the software Colony. The *H*_nb_ per generation and the average number of fathers per generation are indicated in bold.

	*N* (*n*)	*H*_nb_	Multi‐paternity inference		
			a: Locus per locus analysis	b: Multi‐locus analysis	c: Colony
**D_SM1**					
*Generation 4*:		**0.4542**		**6.6**	**6.8**
D_SM1.4.A	37 (17)	0.3729	Yes (1)	9	7
D_SM1.4.B	33 (14)	0.357	0	7	6
D_SM1.4.C	38 (32)	0.4115	0	6	6
D_SM1.4.D	38 (26)	0.3473	0	5	7
D_SM1.4.E	35 (18)	0.3023	Yes (1)	6	8
*Generation 6*:		**0.5276**		**8.3**	**8**
D_SM1.6.A	39 (04)	.	.	.	.
D_SM1.6.B	39 (28)	0.4824	Yes (1)	8	10
D_SM1.6.C	35 (31)	0.4019	Yes (1)	9	8
D_SM1.6.D	35 (23)	0.4664	0	6	7
D_SM1.6.E	38 (20)	0.4497	Yes (1)	10	7
*Generation 8*:		**0.5121**		**7.6**	**7.4**
D_SM1.8.A	40 (34)	0.497	0	8	8
D_SM1.8.B	37 (20)	0.4156	Yes (1)	7	6
D_SM1.8.C	36 (28)	0.4405	0	9	8
D_SM1.8.D	38 (25)	0.4827	0	5	6
D_SM1.8.E	39 (32)	0.4602	Yes (1)	9	9
*Generation 10*:		**0.5068**		**7.6**	**7.2**
D_SM1.10.A	40 (34)	0.3775	Yes (2)	7	7
D_SM1.10.B	39 (39)	0.5051	Yes (1)	8	7
D_SM1.10.C	37 (36)	0.4627	0	8	8
D_SM1.10.D	40 (39)	0.4816	Yes (1)	9	8
D_SM1.10.E	40 (39)	0.359	0	6	6
**Vv_SM1**					
*Generation 4*:		**0.5116**		**7.2**	**6.6**
Vv_SM1.4.A	39 (34)	0.5117	Yes (1)	7	7
Vv_SM1.4.B	40 (33)	0.4769	Yes (1)	7	7
Vv_SM1.4.C	32 (23)	0.4689	0	7	6
Vv_SM1.4.D	38 (27)	0.478	Yes (1)	10	7
Vv_SM1.4.E	40 (32)	0.3319	Yes (1)	5	6
*Generation 5*:		**0.478**		**5.2**	**6.4**
Vv_SM1.5.A	40 (34)	0.3811	0	7	7
Vv_SM1.5.B	38 (31)	0.4194	0	2	5
Vv_SM1.5.C	34 (23)	0.4099	0	7	6
Vv_SM1.5.D	40 (36)	0.4208	0	6	7
Vv_SM1.5.E	40 (36)	0.3111	0	4	7
*Generation 6*:		**0.536**		**7.2**	**6.8**
Vv_SM1.6.A	39 (30)	0.5044	Yes (1)	10	8
Vv_SM1.6.B	39 (39)	0.4173	0	3	4
Vv_SM1.6.C	40 (31)	0.4821	Yes (1)	9	8
Vv_SM1.6.D	39 (33)	0.4713	Yes (1)	8	9
Vv_SM1.6.E	38 (36)	0.4225	Yes (1)	6	5
*Generation 7*:		**0.5219**		**6.6**	**5.6**
Vv_SM1.7.A	40 (31)	0.4864	Yes (1)	7	6
Vv_SM1.7.B	42 (32)	0.4365	Yes (3)	8	6
Vv_SM1.7.C	46 (40)	0.3729	0	7	8
Vv_SM1.7.D	44 (43)	0.5173	0	4	4
Vv_SM1.7.E	39 (34)	0.5248	Yes (2)	7	4
*Generation 8*:		**0.5375**		**6.5**	**5.8**
Vv_SM1.8.A	45 (38)	0.4304	0	5	5
Vv_SM1.8.B	40 (38)	0.3777	0	9	8
Vv_SM1.8.C	35 (13)	.	.	.	.
Vv_SM1.8.D	36 (27)	0.5013	0	4	4
Vv_SM1.8.E	40 (35)	0.4924	0	8	7
*Generation 9*:		**0.5359**		**7.6**	**7.4**
Vv_SM1.9.A	40 (32)	0.471	Yes (2)	7	8
Vv_SM1.9.B	40 (29)	0.5415	Yes (1)	9	7
Vv_SM1.9.C	39 (36)	0.467	Yes (1)	7	8
Vv_SM1.9.D	38 (31)	0.4324	0	9	6
Vv_SM1.9.E	40 (37)	0.3136	Yes (1)	6	8
*Generation 10*:		**0.5201**		**9.4**	**8.6**
Vv_SM1.10.A	40 (38)	0.4357	Yes (1)	10	8
Vv_SM1.10.B	38 (32)	0.5328	Yes (1)	8	8
Vv_SM1.10.C	39 (33)	0.3816	Yes (1)	9	11
Vv_SM1.10.D	38 (28)	0.497	Yes (1)	8	9
Vv_SM1.10.E	40 (39)	0.4663	Yes (1)	12	7

Using the set of six microsatellite markers, we identified 21 alleles among the 2135 *G. pallida* individuals which were genotyped, ranging from two (for Gp117 and Gp122) to six (for Gp108) alleles per locus. Three alleles were found for Gp126 and four alleles were found for Gp116 and Gp112.

A high genetic diversity, with an unbiased expected heterozygosity (*H*_nb_) higher than 0.50, was observed within the two lineages. These *H*_nb_ values are equivalent to the ones estimated in Saint‐Malo populations sampled at the plant scale, which varied between 0.50 and 0.53 (Montarry et al. 2015). The genetic diversity appears equivalent between the two lineages (0.51 for D_SM1 and 0.53 for Vv_SM1). Regarding each generation independently, the unbiased expected heterozygosity (*H*_nb_) ranged from 0.45 to 0.53 for the avirulent lineage and from 0.48 to 0.54 for the virulent lineage.

### 3.2 Analysis of Paternity

A total of 53 cysts were genotyped. For the initial approach, locus per locus inference, maternal genotypes were inferred from alleles observed in the offspring and the number of additional alleles per locus was deduced. This simple approach relies on the fact that the detection of at least three non‐maternal alleles indicates the presence of DNA from more than one male. Only three loci, Gp108 (six alleles), Gp112 (four alleles) and Gp116 (four alleles) have at least four alleles identified in the entire SM population which is low to investigate paternity. However, we found evidence for multiple paternity for 30 females as more than two non‐maternal alleles occurred in at least one of these three loci in their offspring (Table 1).

The number of combinations of alleles from fathers was then inferred to estimate a minimum number of fathers needed to explain the multi‐locus genotype diversity observed in the offspring of each female. Based on this analysis, the manual multi‐locus analysis, all cysts showed multiple paternity. The analysis using the software Colony confirms this result and identify multiple paternity for all cysts. The 53 maternal genotypes deduced in Colony are identical to those deduced in the manual multi‐locus approach. We have tested several sets of allele frequencies, estimated at different scales (cyst, generation, population) (data not show). The number of inferred fathers contributing to the offspring contained within a cyst varied for some cysts, but it was roughly similar. The average number of fathers inferred by Colony across the five cysts within each generation remained unchanged, regardless of the allele frequencies provided to Colony. We then decided to retain the number of fathers determined with the Colony programme with the allele frequencies set calculated at each generation (i.e., J2 of each cyst being merged) (Table 1).

A comparison of the two methods (manual multi‐locus analysis and Colony multi‐locus analysis) using a paired *t*‐test showed that the number of inferred fathers did not differ significantly between them (*t* = 1.188, df = 52, *p* = 0.240). Due to its advantages and greater accuracy, we ultimately chose to retain the minimum number of fathers estimated by the Colony software.

A minimum of four to 11 fathers contributing to the offspring contained within a cyst were deduced by Colony. The average number of fathers estimated at each generation ranged from 5.6 fathering males for cysts of Vv_SM1.7 to 8.6 for cysts of Vv_SM1.10. Either the virulent or the avirulent females had mated with an average of seven males (7.31 and 6.79, respectively).

**FIGURE 2:**
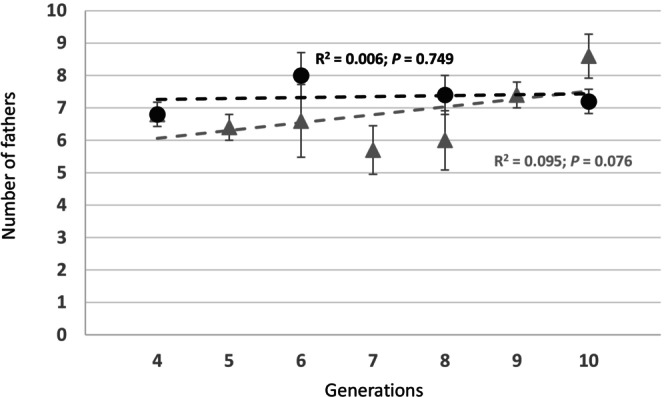
Average number of fathers inferred in each generation when the nematode is reared on the susceptible potato cultivar Desiree (D_SM1, avirulent lineage, black circles) or on the resistant potato cultivar Iledher (Vv_SM1, virulent lineage from different stages of the experimental evolution, grey triangles). The mean and standard error were calculated from the minimum number of fathers inferred by Colony. *R* ^2^ and *p*‐values of the Pearson linear correlations are indicated on the graph.

To explore the polyandry evolution during the adaptation process to the potato resistance, the generation effect was tested for each lineage. Results showed that there was no significant generation effect neither on the avirulent lineage (*F*_3,15_ = 0.879; *p* = 0.474) nor on the virulent lineage (*F*_6,27_ = 2.061; *p* = 0.092). At contrario to our initial expectation, this result showed that the minimum number of fathers did not decrease over generations during the adaptation of the lineage reared on the resistant potato cultivar. Moreover, tests of correlation were also not significant neither for the avirulent nor for the virulent lineage (Figure 2).

The minimum number of fathers was also similar between the two lineages. Indeed, there was no significant lineage effect at each generation (F_1,8_ = 0 and *p* = 1 at the fourth generation; *F*_1,7_ = 0.977 and *p* = 0.356 at the sixth generation; *F*_1,7_ = 1.773 and *p* = 0.225 at the eighth generation; *F*_1,8_ = 3.267 and *p* = 0.108 at the tenth generation). So, the status of the host plant, resistant or susceptible, did not influence the level of polyandry.

To compare these results using population from experimental evolution with natural populations, we have estimated using the same set of microsatellite markers the minimum number of fathers in the offspring of four cysts collected directly from two French field populations (Saint‐Malo and Noirmoutier) that were previously investigated in terms of origin of the heterozygote deficit (Montarry et al. 2015). The minimum number of fathers contributing to the offspring contained within a cyst inferred for these two natural populations ranged from six to nine (Supporting Information S1) and was thus similar to the results obtained with the lineages from experimental evolution.

## 4 Discussion

Microsatellite genotypes were shown to be useful for estimating the relationships and relatedness between PCN individuals (Plantard et al. 2008; Boucher et al. 2013; Blacket et al. 2019; Thevenoux et al. 2019; Handayani et al. 2020; Esquibet et al. 2024). However, additional studies also employ highly polymorphic microsatellite markers to assess mating systems and polyandry in nematodes (Taylor et al. 2014). Recent studies have used microsatellite markers to monitor and validate single pair mating crosses in the ovine parasitic nematode *Haemonchus contortus* (Sargison et al. 2018), to detect hybridization events between lines of the dagger nematode *Xiphinema index* (Nguyen et al. 2021), and to identify admixed lineages of the entomopathogenic nematode *Deladenus siricidicola* (Fitza et al. 2022). Microsatellite markers developed for *G. pallida* , which show a weak prevalence of null alleles (Montarry et al. 2015), provide a powerful tool for screening offspring and conducting parentage analyses. Furthermore, the amplification of microsatellite markers by multiplex PCR can be performed on DNA extracted from a single juvenile, which is advantageous compared with methods such as whole‐genome sequencing, which require a substantial quantity of pure DNA and are currently performed on pools of nematode individuals. In this study, we used a set of six of these microsatellite markers and revealed evidence of multiple paternity in *G. pallida* , clearly highlighting polyandry in this species.

The limited polymorphism of the markers used and the high level of inbreeding (Picard et al. 2004; Montarry et al. 2015) that characterises *G. pallida* populations may together result in an underestimation of the number of fathers. There is thus a possibility that some genetically closely related fathers cannot be differentiated by our multilocus microsatellite genotyping approach. Furthermore, the study was based on a European population maintained under controlled conditions, and therefore exhibiting low genetic diversity. When comparing the results obtained using populations from experimental evolution with those obtained using natural populations, we got a similar number of fathers, suggesting that the loss of genetic diversity does not appear to affect paternity analysis. Whatever the used population, it should be underlined that our approach estimated a minimum number of fathers, rather than a number of fathers, leaving open the possibility that they could be more.

Although polyandry is a common reproductive strategy among many animal species, the frequency of polyandrous female in a population ranged from 0% to 100% (Taylor et al. 2014). In our study, as evidenced by multiple paternity, 100% of *G. pallida* females were polyandrous. This suggests that polyandry is an important feature of this cyst nematode, which should confer benefits (directly or indirectly) in terms of biological fitness. Until now, no obvious directs benefits that immediately improve their survival or fecundity have been reported for *G. pallida* females. Then, in the assumed absence of direct benefits, one explanation for polyandry is that females of *G. pallida* gain indirect genetic benefits for their offspring (Jennions and Petrie 2000). By multiple mating, females are thought to obtain male genes that are compatible with their own genes (Tregenza and Wedell 2002), or ‘good genes’ that enhance the viability or competitiveness of their offspring (Yasui 1998). Moreover, females could benefit from polyandry by accruing male genes that could enhance their offspring's genetic diversity (Yasui 1998; Yasui and Garcia‐Gonzalez 2016). All these hypotheses depend on the predictability of the environment, the ability of females to identify a particular male, based on pre‐copulatory or post‐copulatory cues and ultimately depend on the female mate choice (Yasui and Garcia‐Gonzalez 2016). Sometimes females cannot identify the best male or assess the environmental quality, and under such circumstances, selection may favour females that mate with different males to hedge their bets (Jennions and Petrie 2000; Yasui and Garcia‐Gonzalez 2016). This bet‐hedging strategy via polyandry has for example been empirically demonstrated in the red flour beetle *Tribolium castaneum* (Matsumura et al. 2021).

It seems unlikely that females of *G. pallida* can assess the genetic quality of individual males before mating. The passive acceptance of multiple mating by *G. pallida* females could be a way to compensate the negative effects of consanguinity. Adult cyst nematodes generally mate on the plant where they come from. Only males or juveniles actively disperse, but only over short distances (Wallace 1968). This restricted dispersal ability, which may promote mating between siblings, largely explains the heterozygote deficits (*F*_IS_ > 0) observed in most cyst nematode species such as *H. schachtii* (Plantard and Porte 2004; Kim et al. 2018), *H. glycines* (Wang et al. 2015), and *H. carotae* populations (Gautier et al. 2019; Esquibet et al. 2020). Positive *F*_IS_ seems to be due to consanguineous mating in the monovoltine species *G. pallida* (Montarry et al. 2015). If polyandrous *G. pallida* females do not reduce to zero the risk that all their offspring are sired by close relatives, they increase the probability of mating with at least one genetically different male. Polyandry may thus help to offset the negative effects of consanguinity, but further studies will be needed to test this hypothesis.

Although multiple mating generally increases the fitness of both males and females, polyandry is only beneficial for a female up to a certain point. Mating inevitably *has* costs. For instance, a consequence of mating for *Caenorhabditis elegans* hermaphrodites is physical cuticle damage owing to the act of copulation itself (Woodruff et al. 2014). Increasing the number of matings, beyond approximately five to seven, induces mortality in females of *C. remanei* (Diaz et al. 2009). The minimum number of matings inferred in our study for *G. pallida* females, ranging from four to 11 with an average of seven fathering males, exceeds this optimal mating number identified for *C. remanei*. This suggests that the risk of injury due to male harm would be lower for *G. pallida* females.

Having multiple mates raises questions about the contribution of each male to the next generation. Our results showed that on average seven males contribute to the offspring, but it remains unclear whether they transmit their alleles equally or whether post‐copulatory mechanisms like sperm selection or competition influence paternity outcomes. Indeed, male contributions to the offspring could be unequal. Looking more in depth at the Colony outputs, it appeared that some males produce only a single genotyped offspring within a cyst, whereas up to half of the genotyped offspring (48%) may be attributed to a single male (Supporting Information S2). The mechanisms that bias paternity, such as cryptic female choice, sperm limitation, and sperm competition, are well documented in several taxa. In nematodes, studies mainly focus on the Rhabditidae family and the model organisms *C. elegans* or *C. remanei* (e.g., Kasimatis et al. 2002). We are only beginning to perceive whether these closely related mechanisms operate in PCN. Apparently, no copulation plug that would prevent mating was formed during the reproductive period. Based on our results, we infer that the female spermatheca can at least partially store sperm from up to 11 males; but the sperm competition hypothesis has not yet been explored. According to Green et al. (1970), the fecundity of *G. pallida* females would not depend on the number of males that inseminated them; changing the number of males did not usually alter the average fecundity (eggs/fecund female). This suggests that the costs and benefits of multiple mating may lead *G. pallida* females to mate at a high level.

We have demonstrated here that all *G. pallida* females are polyandrous. Without attempting to fully explain the potential benefits that females may gain from mating more than once, our second objective was to determine whether the number of matings varies over time during the adaptation process to a resistant cultivar (here Iledher) and potentially play a role in the adaptation of *G. pallida* populations. Indeed, a male‐skewed adult sex ratio is expected to increase the frequency of multiple paternity (Székely et al. 2014), females having more opportunities to mate with multiple males. Since masculinising resistance leads to an unequal sex ratio in *G. pallida* avirulent populations, we expected to observe higher frequencies of polyandry in females from an avirulent population than in those from a lineage adapted to the GpaV_vrn_ QTL when the sex ratio becomes less male‐biased. Our results showed that there was no significant generation effect either on the avirulent lineage or on the virulent lineage. Moreover, there was no significant lineage effect at each generation. An average of a minimum number of seven fathering males was identified all along the adaptation process to a masculinizing resistance.

A simple explanation for the absence of variation of the minimum number of fathers across the adaptation process is the possibility that the number of fathers was higher during the first three generations, with the expected decline occurring before the fourth generation. The number of mates estimated from the fourth generation onwards would therefore be at the same level as that of Désirée. We were unable to estimate polyandry during the first three generations. The number of cysts obtained on Iledher during these first generations of the experimental evolution was low and entirely used for subsequent multiplications. However, this hypothesis, which cannot be excluded, would require an even greater number of fathers and therefore of mating events than those observed.

Multiple mating, as evidenced by the minimum number of fathers, appears to remain stable at around seven, regardless of the number of males present around the female. This means that, when the population is female‐biased, under favourable conditions such as on a susceptible host, there are sufficient males to sustain both a high frequency of polyandrous females and a maximum number of matings. This also suggests that males are able to move sufficiently to fertilise several females and that females remain attractive long enough to mate with multiple males. Since both the frequency of polyandrous females and the number of matings already reach their maximum when the population is female‐biased, no further increase is observed when the population is male‐biased, such as in the presence of a cultivar with resistance to *G. pallida* for an avirulent population.

The huge majority of European potato‐resistant cultivars come from *S. vernei* , meaning that resistance is masculinizing and virulence is recessive. A virulent monoandrous female (homozygote vir/vir at the virulence locus) reared on potato‐resistant cultivars would thus produce a 100% virulent offspring if she mates with a virulent male (vir/vir). But this female would also produce either a 50% virulent offspring if she mates with an avir/vir heterozygote male (phenotypically avirulent) or a 100% avirulent offspring if she mates with a homozygous avir/avir male (phenotypically avirulent). On the one hand, polyandry could thus spread the risk of mating only with a homozygote avirulent male. High frequencies of polyandry increase the likelihood that virulent females will mate with at least one male carrying the virulence allele. On the other hand, polyandry would also increase the probability of maintenance of the avirulence allele in the population, and this will play an important role in the durability of masculinizing resistances. Polyandry would be an important parameter to consider in potential future development of realistic models, but as the number of mates seems stable during the adaptation process, it would not change the outputs of the recently developed demo‐genetic model designed to simulate over multiple years the dynamic of the nematode adaptation (Tankam‐Chedjou et al. 2024).

## Author Contributions

**Magali Esquibet:** formal analysis (equal), investigation (equal), writing – original draft (equal). **Océane Lechevalier:** conceptualization (equal), writing – review and editing (equal). **Nathan Garcia:** investigation (equal), writing – review and editing (equal). **Sylvain Fournet:** investigation (equal), writing – review and editing (equal). **Eric Grenier:** conceptualization (equal), writing – review and editing (equal). **Josselin Montarry:** conceptualization (equal), formal analysis (equal), funding acquisition (equal), investigation (equal), writing – original draft (equal).

## Funding

This work was supported by HORIZON EUROPE European Innovation Council, NEM‐EMERGE no. 101083727.

## Conflicts of Interest

The authors declare no conflicts of interest.

## Supporting information


**Supporting Information: S1** Number of genotyped individuals (*N*) and number of individuals without missing data (*n*) per cyst and minimum number of fathers per cysts as estimated by (a) locus per locus analysis: initial multi‐paternity inference and (number of loci with three or more non‐maternal allele), (b) multi‐locus analysis: minimum number of fathers based on the number of multi‐locus genotype combinations, (c) Colony: number of fathers based on the number of multi‐locus genotypes inferred using the software Colony.

**Supporting Information: S2a** Number of genotyped individuals (*N*) and number of individuals without missing data (*n*) per cyst Vv_SM1, number of fathers per cysts and full‐sib families inferred by the software Colony. The contribution of males to offspring for each family is expressed as a percentage (number of offspring/*N*). The maximum number of offspring observed in a family is shown in bold. The minimum number is shown in grey and bold. The inclusive and exclusive probabilities of each family are not included in the table. Note that they are generally low, indicating that the family can be split into more families. This is why we refer to a minimum number of males.

## Data Availability

The file (Esquibet_Polyandry_Gp.txt) containing the genotypic data (Genepop format) for the 55 cysts of *Globodera pallida* is available as a File S1.
