# Supplementary material for: First Insight Into Multiple Paternity in the Potato Cyst Nematode Globodera pallida Facing Masculinizing Plant Resistance
Source: Ecol Evol. 2026 Jun 29;16(7):e73885. doi: 10.1002/ece3.73885 (PMC13314386; doi:10.1002/ece3.73885)
Supplement: Supplementary file 1 — Supporting Information: S1 Number of genotyped individuals (N) and number of individuals without missing data (n) per cyst and minimum number of fathers per cysts as estimated by (a) locus per locus analysis: initial multi‐paternity inference and (number of loci with three or more non‐maternal allele), (b) multi‐locus analysis: minimum number of fathers based on the number of multi‐locus genotype combinations, (c) Colony: number of fathers based on the number of multi‐locus genotypes inferred using the software Colony. [file ECE3-16-e73885-s002.docx]

**Supporting Information S1 |** Number of genotyped individuals (N) and number of individuals without missing data (n) per cyst and minimum number of fathers per cysts as estimated by **a)** locus per locus analysis: initial multi-paternity inference and (number of loci with three or more non-maternal allele), **b)** multi-locus analysis: minimum number of fathers based on the number of multi-locus genotype combinations, **c)** Colony: number of fathers based on the number of multi-locus genotypes inferred using the software Colony.

|  | N (n) | **a:** locus per locus analysis | **b:** multi-locus  analysis | **c:** Colony |
| --- | --- | --- | --- | --- |
| **SM08**  SM08.13  SM08.14  **N2_02**  N2_02_M4  N2_02_M5 | 40 (40)  40 (40)  37 (34)  36 (36) | Yes (1)  Yes (1)  Yes (1)  Yes (2) | 11  11  11  5  3  7 | 8  **7**  9  6  6  6 |
